# Clinical-Pathological Evaluation and Prognostic Analysis of 228 Merkel Cell Carcinomas Focusing on Tumor-Infiltrating Lymphocytes, MCPYV Infection and ALK Expression

**DOI:** 10.1007/s12022-022-09716-2

**Published:** 2022-05-12

**Authors:** Federica Santoro, Francesca Maletta, Renato Parente, Jessica Fissore, Cristian Tampieri, Leonardo Santoro, Nadia Birocco, Franco Picciotto, Pietro Quaglino, Marco Volante, Sofia Asioli, Rebecca Senetta, Mauro Papotti

**Affiliations:** 1 Pathology Unit, Department of Medical Sciences, University of Turin, Turin, Italy; 2 Pathology Unit, Department of Laboratory Medicine, Città Della Salute e Della Scienza of Turin, Turin, Italy; 3 Pathology Unit, Humanitas-Gradenigo Hospital, Turin, Italy; 4 Pathology Unit, Department of Oncology, Città Della Salute e Della Scienza of Turin, University of Turin, Via Santena 7, 10126 Turin, Italy; 5 Institute of Mathematics (EPFL), Lausanne, Switzerland; 6 Oncology Unit, Città della Salute e della Scienza, Turin, Italy; 7 Dermatologic Surgery Section, Department of Surgery, Città Della Salute e Della Scienza of Turin, Turin, Italy; 8 Dermatology Clinic, Department of Medical Sciences, Città Della Salute e Della Scienza of Turin, University of Turin, Turin, Italy; 9 Pathology Unit, Department of Oncology, San Luigi Hospital of Orbassano, University of Turin, Turin, Italy; 10 Pathology Unit, Department of Biomedical and Neuromotor Sciences (DIBINEM), University of Bologna, Bologna, Italy

**Keywords:** Merkel cell carcinoma, Merkel cell polyomavirus, Tumor-infiltrating lymphocytes, ALK expression, INSM1 expression

## Abstract

Merkel cell carcinoma is a rare and aggressive primary neuroendocrine carcinoma of the skin, whose pathogenesis can be traced back to UV radiation damage or Merkel cell polyomavirus (MCPyV) infection. Despite some improvements on the characterization of the disease partly due to its increased incidence, crucial pathogenetic and prognostic factors still need to be refined. A consecutive series of 228 MCC from three hospitals in Turin was collected with the aim of both analyzing the apparent increase in MCC incidence in our area and investigating the distribution and prognostic role of clinical-pathological parameters, with a focus on MCPyV status, ALK tumor expression and tumor infiltrating lymphocytes (TILs). Review of morphology and conventional immunohistochemical staining was possible in 191 cases. In 50 cases, the expression of the novel neuroendocrine marker INSM1 was additionally assessed. Fourteen cases of MCC of unknown primary skin lesion were identified and separately analyzed. While confirming an exponential trend in MCC incidence in the last decades and providing a description of histological and cytological features of a large series of MCC, the present study concludes that 1) INSM1 is a highly sensitive marker in both skin and lymph node primary MCC; 2) positive MCPyV status, brisk TILs and lower tumor size and thickness are independent positive prognostic parameters, and the combination of the former two may provide a novel tool for prognostic stratification; 3) ALK is expressed 87% of MCC and associated with positive viral status, and could represent a prognostic biomarker, if validated in larger series.

## Introduction

Merkel cell carcinoma (MCC) is a rare aggressive primary neuroendocrine carcinoma of the skin, most frequently affecting elderly and fair-skinned individuals and favored by immunosuppression [1–3]. Although traditionally considered a rare disease, the evident increase of incidence observed in the last decades led to a growing interest in its origin and treatment [4–8]. The pathogenesis of MCC can be traced back to two independent pathways: UV radiation induced damage and Merkel cell polyomavirus infection (MCPyV), which define two genetically distinct subgroups. UV-induced tumors have a very high mutational burden, with frequent loss of function mutations of RB and TP53 genes, activating mutations of PIK3CA, and MYC amplification [1]. Virus-induced tumors are defined by a low tumor mutational burden and MCPyV DNA integration resulting in viral oncoproteins expression [9, 10]; this subgroup represents at least 60% of all MCC [1] (up to 80% in the Northern hemisphere) [11]. The two different pathogenetic mechanisms seem to reflect diverse clinical outcomes: UV-induced tumors are associated with a higher risk of recurrence, progression and death [12], whereas MCPyV-related tumors show better outcome, irrespective of tumor stage and patient immune status [13].

Prognostic evaluation of MCC traditionally relies on tumor size, lymph node involvement, and distant metastases [14]. In the last years, however, further clinical and morphological features, such as sex and age, immunosuppression, as well as tumor-infiltrating lymphocytes (TILs) and tumor thickness, have been investigated in their prognostic value [15–19]. In addition, cKIT, PDGFRA, ALK and EGFR represent only some of the biomarkers examined in MCC in order to better define its pathogenesis [20–25]. In particular, ALK tumor expression has been reported in MCC and was recently shown to correlate with viral status as well as a longer survival [20, 22, 26].

Analyzing a consecutive series of MCC from three centers in Turin, the aim of our study was twofold: i) to investigate the prognostic/predictive role of MCPyV status, ALK tumor expression and clinical-pathological parameters in respect to patient’s progression and outcome, and ii) to analyze and confirm the apparent increase in MCC incidence in the last decades.

## Materials and Methods

### Subjects and Samples

A retrospective series of 228 cases of MCC diagnosed between January 1980 and July 2021 was collected from three main centers in Turin: specifically, 193 cases from Città della Salute e della Scienza Hospital, 24 cases from Humanitas Gradenigo Hospital and 11 cases from San Luigi Gonzaga Hospital**.** Città della Salute e della Scienza University Hospital’s cases included consultation cases from local hospitals of the city of Turin and from outside provinces, since the hospital acts as a reference center for neuroendocrine pathology. Histological slides were independently reviewed by three different pathologists with an interest in skin and neuroendocrine pathology (FS, RS, MP), all blinded to the patient’s outcome. Tumor formalin-fixed paraffin-embedded (FFPE) blocks and/or original histological slides were available for 191 patients (84%); of those, 144 were skin biopsy samples (17 incisional and 127 excisional, 34 of which had an associated positive lymphadenectomy) and 47 were lymph node or visceral localizations for which no cutaneous sample was available. In the remaining 37 cases, material was either not available or returned when received as a second opinion request. Archival immunohistochemical slides of each case were reviewed and included, apart from conventional hematoxylin and eosin stains, either a broad-spectrum keratin or a cytokeratin 20 (CK20) stain and additional stains for chromogranin A (CGA, 156 cases), synaptophysin (100 cases) and Ki67 (97 cases). Few older cases included stains for neuron-specific enolase (NSE) and neurofilaments. Diagnosis was confirmed in all cases based on morphological and immunohistochemical features, with positive expression of a broad spectrum keratin or CK20 with a dot-like paranuclear pattern, combined with at least one neuroendocrine marker [11]. The most representative slide of each sample was selected for additional immunohistochemical tests, in cases with available material. Clinical data and patient identification were anonymized prior to analysis by a pathology staff member not involved in the study.

### Clinical and Follow-up Data and Morphological Evaluation

Clinical data were retrospectively obtained from medical records. Our analysis included sex, age at diagnosis, tumor size and location, date of histological diagnosis, date of disease progression and/or death (MCC-related or not). Loco-regional recurrence or distant metastases were documented. Tumor staging was re-assigned according to the AJCC 8th edition TNM Staging system [27].

Neoplastic growth pattern (trabecular/organoid versus diffuse), the extent of neoplastic necrosis and the cytological features of tumor cells were assessed on cases for which a primary skin sample or a lymph node or visceral localization was available. Cell type was classified as conventional (i.e., round and small sized nuclei with salt and pepper chromatin) versus spindled (slightly pleomorphic cells with spindle-shaped nuclei and vaguely fascicular pattern) versus large (larger nuclei and inconspicuous cytoplasm) versus pleomorphic (comprising scattered very large pleomorphic or multinucleated cells). Breslow thickness [28], presence of ulceration, Clark level [28], presence of deep extradermal invasion, deep tumor edge (pushing versus infiltrating), mitotic rate (as number of mitoses/mm2), vascular and perineural invasion, evidence of heterologous differentiation, solar elastosis in adjacent dermis [28], margins status and inflammatory infiltrate (absent versus present non-brisk versus present brisk) [28] were assessed on primary skin samples only. Specifically, tumors with brisk TILs included tumors with diffuse infiltrates both at the tumor periphery and within the tumor stromal septa.

### Immunohistochemical Procedures

Three micron-thick serial paraffin sections of each case were processed by immunohistochemistry using an automated immunostainer (Ventana BenchMark AutoStainer, Ventana Medical Systems, Tucson, AZ, USA) with antibodies against INSM1 (clone A8, diluted 1:100, Santa Cruz Biotechnology, Inc., Dallas, Texas, USA), MCPyV large T-antigen (Mouse Monoclonal Antibody, clone CM2B4, diluted 1:50; Santa Cruz Biotechnology, Inc., Dallas, Texas, USA) and ALK (Rabbit Monoclonal Primary Antibody, clone D5F3, pre-diluted; Roche Diagnostics, Inc., Tucson, AZ, USA). Appropriate positive controls were included for each immunohistochemical run. Immunohistochemical staining was interpreted by two pathologists (FS and RS). INSM1 expression was evaluated in 50 cases. MCPyV status was assessed in 178 cases and interpreted according to Moshiri et al. [12]; cases were considered negative when weak staining was observed in < 1% of tumor cells. ALK staining was performed in 155 cases and scored according to both the percentage of positive tumor cells and their staining intensity as negative, low, intermediate or high (partially based on previous literature data [20, 26, 29]). Specifically, the reaction was considered negative when staining was observed in < 1% of tumor cells; low when very weak to moderate staining intensity was observed in < 25% of tumor; intermediate when weak to moderate staining intensity was observed in > 25% of tumor cells, or when strong staining intensity was observed in < 25% of tumor cells; high when strong staining intensity was observed in > 25% of tumor cells.

### Statistical Analyses

Data were collected using Microsoft ® Excel 2020 software and analyzed with Python3 programming framework. Associations between clinical-pathological variables were investigated by means of Student *t*-tests and Pearson's Chi-squared tests. Due to technical issues in the execution and evaluation of the MCPyV large T-antigen immunohistochemistry in older and consequently less well-preserved cases, we decided to focus our statistical analyses concerning viral status and its correlation with clinical-pathological features on cases diagnosed from the year 1990. Differences in clinical-pathological parameters with respect to viral status and patient’s progression were separately explored by means of Student *t*-tests and Pearson’s Chi-squared tests. Statistical significance for individual regression coefficients—and hence the (adjusted) odds ratios—was tested using likelihood ratios. Survival rates for Disease Specific Survival (DSS) and Disease Free Interval (DFI) were estimated by Kaplan–Meier method. DSS was calculated as the number of months from the date of histological diagnosis to the date of death by MCC or of last follow-up. DFI was calculated as the time elapsed from the date of histological MCC diagnosis to the date of first evidence of disease progression or of the last follow up, considering only the patients in which the treatment performed at diagnosis had rendered them free of disease. To investigate covariation of clinical and pathological parameters and survival rates, a Cox’s proportional hazard model was fitted to the data, both in univariate and multivariate setting. Variable selection for the multivariate analysis was based on both univariate significance and prior knowledge [30]. Statistical significance was set at *p* < 0.05.

To evaluate the increase in MCC diagnoses in the population of Turin, both a Linear Model (LM) and a Generalized Linear Model (GLM) regression analysis were fitted to the yearly number of diagnosed cases per million inhabitants between the years 1980 and 2021 (Italian Institute of Statistics data). For the purpose of a precise incidence analysis limited to the city of Turin, 35 cases that arrived as second opinion request from hospitals outside the city of Turin were excluded. Statistical significance for individual regression coefficients was tested using likelihood ratios, and confidence intervals were computed by the asymptotic normality of the maximum likelihood estimated coefficients. The dynamics of such increase were further explored by means of an Age-Period-Cohort model (APC), accounting for the age (A), date of diagnosis (P) and date of birth (C) of patients. Average counts of diagnoses were aggregated in 5-years periods. Due to the significant limitations posed by the application of such analyses to our relatively small sample, the APC analysis was performed exclusively as an exploratory analysis assessing whether our results were consistent with larger cases studies in literature.

## Results

### Patient’s Characteristics and Follow-up Data

Our retrospective cohort included 228 patients, 123 males (54%) and 105 females (46%). The patients’ age ranged from 38 to 102 years, with a mean age of 74.5 years. Cutaneous tumor size ranged from 0.3 to 14 cm (mean 2.7 cm). Primary skin tumor site was retrieved from medical records for 196 cases: the majority were located on the extremities (90, 46%) followed by head and neck regions (68, 35%), buttocks (26, 13%), and trunk (12, 6%). Of the remaining cases, 18 lacked this information, whereas 14 (6%) exhibited an exclusive lymph node localization for which no primary cutaneous lesion was found, despite thorough clinical-instrumental evaluation (MCC of unknown primary, MCC-UP). Tumor stage was re-assessed in 192 patients, of which 126 (66%) were stage I-II (indicating a localized primary skin tumor), 60 (31%) were stage III with loco-regional lymph node spread and 6 (3%) were stage IV, indicating the presence of visceral metastases. Stage was not assigned in MCC-UP patients and in those with incomplete information (22 cases). Follow-up (FU) data were available for 195 (85%) cases. FU ranged from 1 to 214 months with a mean duration of 36.34 months. Disease progression was observed in 65 cases. At the end point of FU, 112 out of 195 patients (57%) were alive, of which 86 (77%) with no evidence of disease and 26 (23%) alive with disease; 83 patients (43%) had died, either of disease (61, 73%) or due to other causes (22, 27%). Table 1 details patient’s demographics, clinical findings and outcome of the present series.

**Table 1:** Summary of patient’s demographics, clinical-pathological parameters, and outcome of Merkel cell carcinoma cases

*Characteristic*		*Number of patients (%)*
Sex	Male Female	123 (54%) 105 (46%)
Age (years)		Mean: 74.5
Primary tumor site	Head and neck Trunk Buttocks Extremities	68 (35%) 12 (6%) 26 (13%) 90 (46%)
Tumor size (cm)		Mean: 2.7
Breslow thickness (mm)		Mean: 11.8
Clark level	III IV V	3 (2%) 13 (9%) 128 (89%)
Deep extradermal invasion	Absent Present	138 (97%) 5 (3%)
Tumor stage (TNM 8^th^ edition)	1 2A 2B 3A 3B 4	65 (34%) 58 (30%) 3 (2%) 8 (4%) 52 (27%) 6 (3%)
Tumor edge	Pushing Infiltrative	98 (69%) 44 (31%)
Growth pattern	Trabecular/Organoid Diffuse	133 (94%) 9 (6%)
Vascular invasion	Absent Present	33 (23%) 111 (77%)
Skin ulceration	Absent Present	96 (67%) 48 (33%)
Necrosis	Absent Focal Moderate Extensive	60 (42%) 46 (31%) 11 (8%) 27 (19%)
Tumor infiltrating lymphocytes	Absent Present (non-brisk) Present (brisk)	39 (27%) 64 (45%) 39 (27%)
Mitoses/mm^2^		Mean: 32
Solar elastosis (sec. WHO)	Absent 1 2 3	21 (16%) 36 (26%) 20 (15%) 58 (43%)
Cell type	Conventional Spindle cell Large cell Pleomorphic	85 (60%) 20 (14%) 35 (24%) 3 (2%)
Viral status	Negative Positive	56 (31%) 122 (69%)
ALK expression	Absent Low Moderate High	20 (13%) 33 (21%) 42 (27%) 60 (39%)
Follow up	NED AWD DOD DOC lost	86 (38%) 26 (11%) 61 (27%) 22 (9%) 33 (15%)

*NED* No Evidence of Disease, *AWD* Alive with Disease, *DOD* Dead of Disease, *DOC* Dead of Other Causes

### Morphological and Immunohistochemical Features of MCC

#### MCC with Known Primary Skin Lesion

Among the 144 cases for which a primary skin sample was evaluable, MCC grew as a dermal malignancy with frequent invasion of the subcutis: Clark level V was recorded in 89% of cases, while 9% of cases had a Clark Level IV and only 2% had a Clark Level III. Breslow thickness ranged from 1.9 to 45 mm (mean 11.8 mm). Skin ulceration was present in 33% of cases. The growth pattern at the tumor edge was more often pushing than infiltrating (69% versus 31%). Vascular embolization was often identified and extensive (77%), whereas perineural invasion was observed in one case, only. TILs were present in 103 cases (73%), of which 39 cases (38%) displayed a brisk pattern. Number of mitotic figures/mm2 ranged from 5 to over 40 with a mean value of 32/mm2. Tumor necrosis was observed in 84 cases (58%). Only two cases displayed focal squamous differentiation, and further two were characterized by prominent glomeruloid vascular proliferations. Epidermotropism and/or MCC in situ [31] were not observed. Tumor cells displayed conventional neuroendocrine-like features in 59% of cases; the remaining cases were further described as large (25%), spindled (14%), and pleomorphic (2%). Solar elastosis was present in the peritumoral dermis in 84% of cases and was recorded as grade 3 in 58 samples. Table 1 summarizes morphological parameters of MCCs included in the study. In 30 cases of MCC, only a nodal sample was available. Among these, tumors showed a trabecular/organoid pattern of growth in 18 cases (60%), whereas necrosis was detected in 83% of tumors (25 cases). Cell type was conventional small cell in 60% of cases (18), spindled in 23% (7) and large in 17% (5) of cases.

#### MCC of Unknown Primary Skin Lesion

In 14 of our cases, MCC was diagnosed as a lymph node disease with no prior or subsequent clinical or radiological evidence of a primary tumor (MCC-UP). Ten patients were males (72%) and 4 females (28%) and the mean age was 65 years. Histologically, the tumor grew with a trabecular/organoid or diffuse pattern with a complete effacement of lymph node architecture in the majority of cases (77%); only few cases displayed a peripheral residual of lymph node tissue. Necrosis was observed in all cases. Tumor cells showed conventional morphology in 70% of cases, whereas 15% were described as spindled and 15% as large. Table 2 reports clinical and histological parameters of MCC-UP.

**Table 2:** Clinical-pathological comparison between MCC-UP and MCC-KP (stage I-II-III)

	**MCC-UP**	**MCC-KP**	***p***
Sex			
Male	10	109	0.269
Female	4	99	
Age (years)	Median: 64	Median: 76	**0.026**
	Mean: 65	Mean: 75	
Lymph node site			
Head and neck	0	13	0.423
Axillary	2	12	0.934
Inguinal	11	38	0.177
Necrosis			
Absent	0	64	**0.047**
Present	13	108	
Cell type			
Conventional	9	103	0.729
Spindle cell	2	27	0.721
Large cell	2	38	0.52
Pleomorphic	0	3	0.611
Viral status			
Negative	1	52	0.123
Positive	12	108	
ALK expression			
Negative	1	18	0.992
Positive	11	121	
Disease progression			
No	5	114	0.494
Yes	5	59	

*MCC-UP* MCC with Unknown-Primary; *MCC-KP* MCC with Known-Primary

#### Immunohistochemical Features

Immunohistochemical profile showed a positivity for broad-spectrum keratin or CK20 with a dot-like paranuclear pattern in 100% of cases; chromogranin A and synaptophysin were expressed in 92 and 95% of stained cases, respectively. Ki67 proliferation index was originally assessed in 97 cases and ranged from 20 to 99% of neoplastic cells (mean value: 71%). 21 cases were also originally found reactive for NSE and/or Neurofilament (the latter with a dot like pattern). In addition, 50 tumors were immunostained for the novel diagnostic marker INSM1, which yielded a predominantly strong and diffuse nuclear reactivity in all analyzed cases: staining was observed in at least 80% of tumor cells in all cases.

### Immunohistochemical Evaluation of MCPyV Status and ALK Expression: Correlation with Clinical and Pathological Parameters

**Fig. 1:**
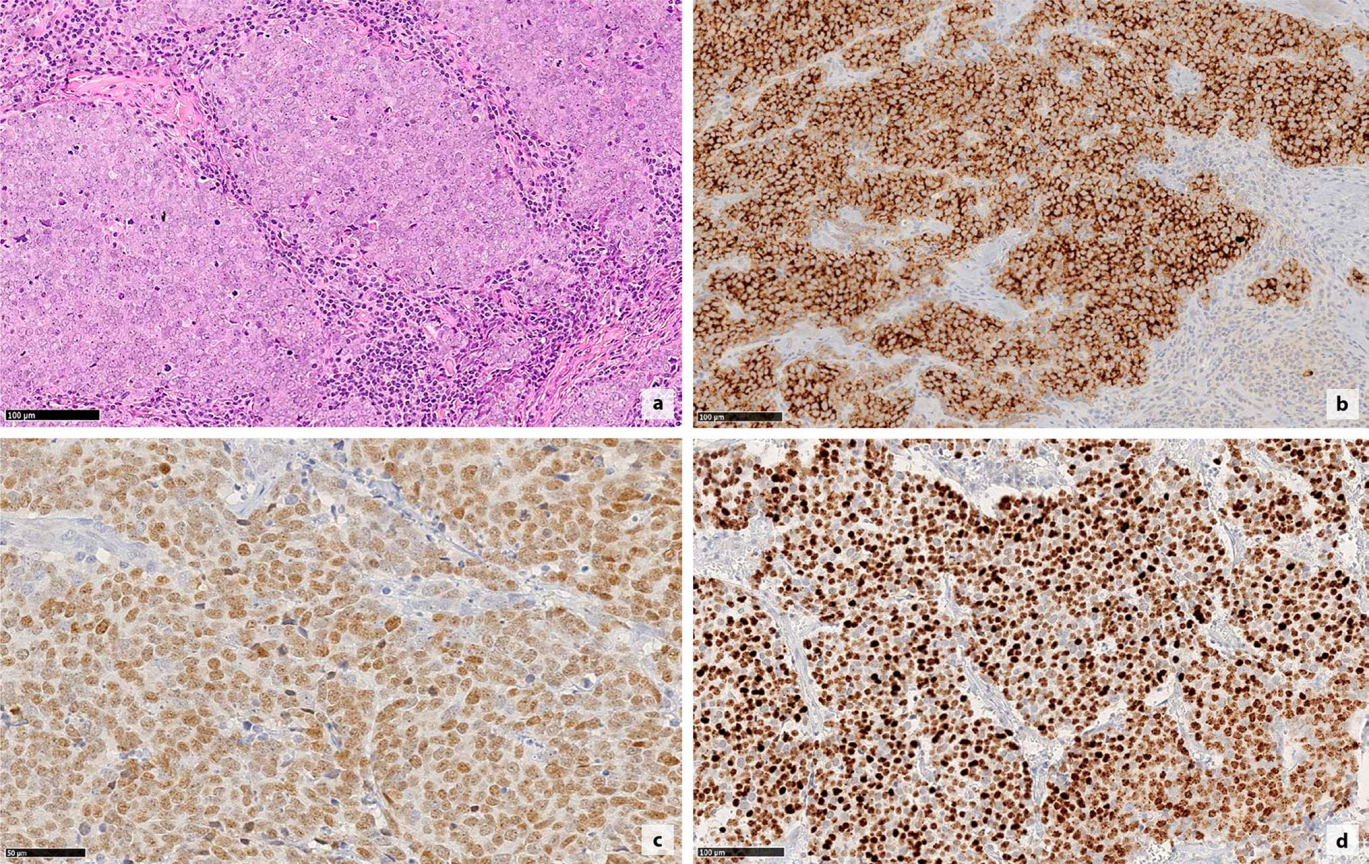
Morphological features of Merkel cell carcinoma with large-cell cytotype and brisk intratumoral tumor infiltrating lymphocytes (**a**), hematoxylin–eosin, magnification 200x). ALK immunohistochemical staining, score 3 (**b**), and MCPyV immunohistochemistry highlighting positive viral status (**c**). INSM-1 immunohistochemistry with strong nuclear expression in the majority of tumor cells (**d**)

MCPyV status was assessed in 178 cases: 69% of cases were MCPyV + and 31% MCPyV- (Table 1 and Fig. 1). Immunohistochemical reaction was prevalent at the slide margin and was very weak in older cases (especially cases diagnosed before the year 1990), suggesting a poor antigen preservation in archival materials. Interestingly, four negative and two positive cutaneous cases displayed focal nuclear MCPyV reactivity in TILs. ALK expression in tumor cells was evaluated in 155 cases and detected in 135 (87%), among which 44% were scored as high, 31% as moderate and 24% as low (Table 1 and Fig. 1).

MCPyV + cases showed a younger age at diagnosis (*p* = 0.001), a lower tumor proliferation index (Ki67, *p* = 0.015) and a lower amount of ulceration (*p* = 0.004) compared to MCPyV- cases. MCPyV + MCCs proved to be relatively more commonly localized in the buttocks (*p* = 0.008), whereas a prevalence of virus-negative cases was observed in MCC localized in the head and neck region (*p* = 0.002). As expected, presence of elastosis and increased severity of elastosis was associated with negative viral status (*p* = 0.042). Cytologically, MCPyV + cases correlated with a large-cell phenotype (*p* = 0.003) while only virus negative cases displayed a pleomorphic phenotype (*p* = 0.041). ALK expression (evaluated as continuous or dichotomized variable) showed a positive correlation with viral status (*p* < 0.001). Table 3 reports the distribution of all clinical-pathological features according to viral status.

**Table 3:** Distribution of clinical-pathological features according to viral status

	***MCPyV status***^a^		
	***Positive***	***Negative***	***p***
Sex			
Male	68	31	0.615
Female	50	18	
Age (years)*	Median: 74	Median: 77	**0.001**
	Mean: 73.36	Mean78.7	
Tumor site			
Head and neck	29	23	**0.002**
Trunk	6	3	0.989
Buttocks	19	0	**0.008**
Extremities	49	14	0.241
Tumor size (cm)*	Median: 2	Median: 2	0.758
	Mean: 2.6	Mean: 2.7	
Breslow thickness (mm)*	Median: 11	Median: 10	0.766
	Mean: 11.8	Mean: 12	
Deep extradermal invasion			
Absent	81	39	0.891
Present	3	2	
Tumor edge			
Expansive	55	31	0.392
Infiltrative	28	10	
Growth pattern			
Trabecular/organoid	92	45	0.147
Diffuse	21	4	
Vascular invasion			
Absent	23	8	0.421
Present	61	34	
Skin ulceration			
Absent	65	21	**0.004**
Present	17	19	
Necrosis			
Absent	44	15	0.449
Present	71	34	
Cell type			
Conventional	62	32	0.238
Spindle cell	14	9	0.423
Large cell	36	4	**0.003**
Pleomorphic	0	3	**0.041**
Tumor infiltrating lymphocytes			
Absent/present non-brisk	60	30	0.912
Present brisk	23	11	
Tumor infiltrating lymphocytes			
Absent	19	12	0.581
Present (non-brisk, brisk)	64	29	
Mitoses/mm2			
Median	26	40	**0.011**
Mean	30	37	
Solar elastosis (0—> 3)*	Median: 2	Median: 3	**0.042**
	Mean: 1.76	Mean: 2.2	
ALK expression			
Negative	4	11	**0.0003**
Positive	96	31	
Proliferation index (Ki67)*	Median: 70%	Median: 80%	**0.015**
	Mean: 68%	Mean: 79%	

*by means of Pearson's Chi-squared or Student t-tests

^a^*evaluated by immunohistochemistry (on cases diagnosed from 1990)*

When comparing clinical and morphological features and ALK expression, we observed a strong correlation between ALK positivity and viral status (*p* < 0.001); no other statistically significant correlations were found.

### Patient’s Progression and Survival Analysis: Correlation with Clinical and Pathological Parameters

Disease progression was significantly associated with male sex (*p* = 0.05), increasing tumor size (*p* = 0.001), deep extradermal invasion (*p* = 0.048), higher tumor stage (*p* = 0.029) and presence of vascular embolization (*p* = 0.05). Table 4

**Table 4:** Distribution of clinical-pathological parameters according to disease progression

	***Disease progression***		
	***Yes***	***No***	***p***
Sex			
Male	42	58	**0.049**
Female	23	62	
Age (years)*)	Median: 74	Median: 75	0.987
	Mean: 73.9	Mean: 73.9	
Tumor site			
Head and neck	18	42	0.665
Trunk	4	7	0.935
Buttocks	11	14	0.295
Extremities	22	49	0.768
Tumor size (cm)*)	Median: 3	Median: 2	**0.001**
	Mean: 3.64	Mean: 2.30	
Breslow thickness (mm)*	Median: 14	Median: 10	0.074
	Mean: 13.90	Mean: 11.21	
Deep extradermal invasion			
Absent	33	84	**0.048**
Present	4	1	
Tumor stage*^a^	Median: 2	Median: 2	**0.029**
	Mean: 3.3	Mean: 2.4	
Tumor edge			
Pushing	25	61	0.728
Infiltrative	12	23	
Vascular invasion			
Absent	4	24	**0.05**
Present	34	61	
Skin ulceration			
Absent	26	60	0.994
Present	11	23	
Necrosis			
Absent	18	39	0.585
Present	38	64	
Cell type			
Conventional	32	58	0.973
Spindle cell	7	15	0.904
Large cell	13	24	0.853
Pleomorphic	1	2	0.588
Tumor infiltrating lymphocytes			
Absent / present non-brisk	28	56	0.279
Present brisk	8	29	
Mitoses/mm2*	Median: 26	Median: 31	0.605
	Mean: 31	Mean: 32	
Viral status			
Negative	13	26	0.84
Positive	42	72	
ALK expression			
Negative	5	10	0.943
Positive	46	75	

*by means of Pearson's Chi-squared or Student t-tests

^a^*Evaluated as a continuous variable considering 1 = stage 1; 2 = stage 2A; 3 = stage 2B; 4 = stage 3A; 5 = stage 3B; 6 = stage 4*

**Fig. 2:**
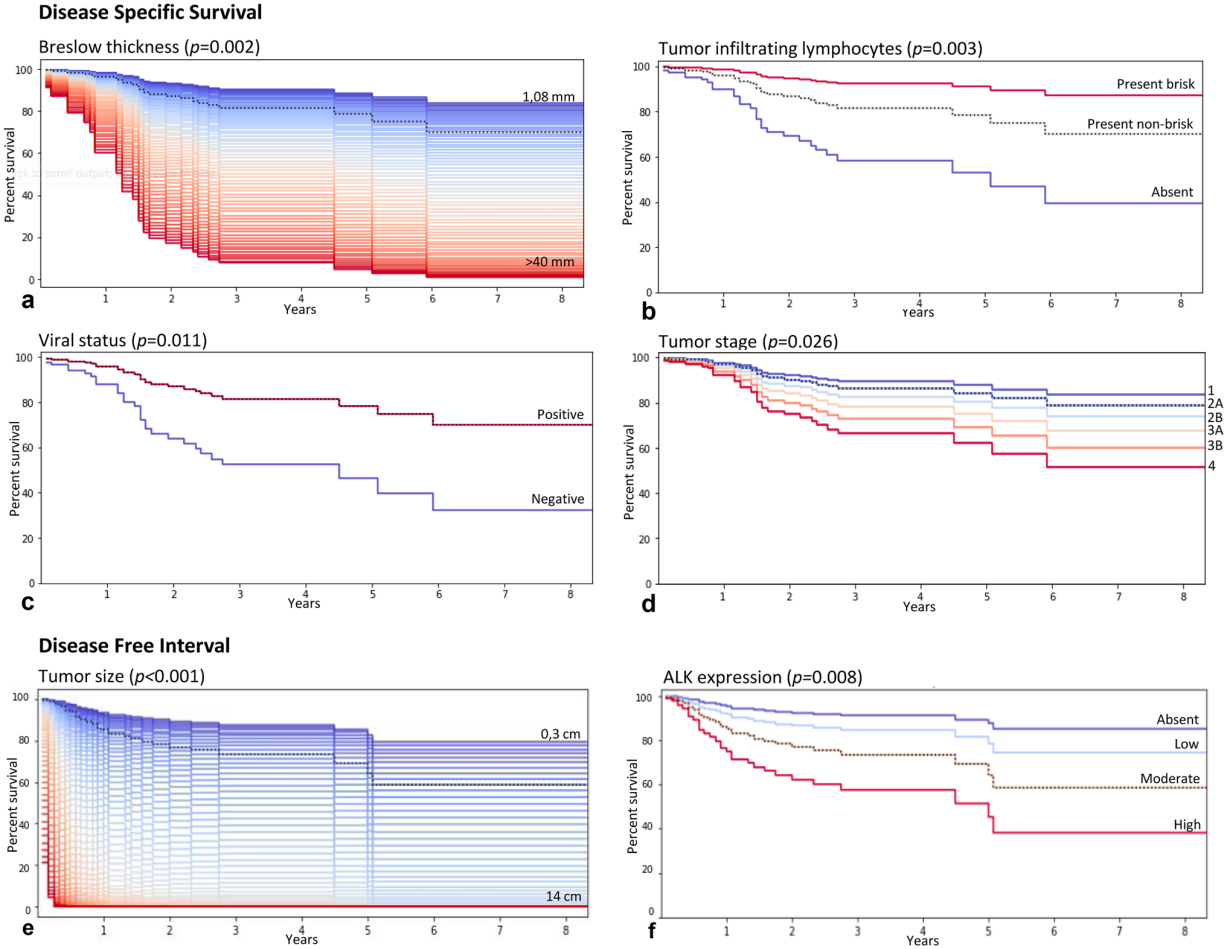
Lower Breslow thickness (**a**), presence of TILs (**b**), positive viral status (**c**) and lower tumor stage (**d**) are associated with longer DSS by multivariate analysis; larger tumor size (**e**) and increased score of ALK immunohistochemical expression (**f**) are associated with shorter DFI by multivariate analysis

By univariate analysis, older age (*p* = 0.01), increased Breslow thickness (*p* = 0.003), tumor size (*p* < 0.001) and higher tumor stage (*p* = 0.002) identified subgroups of patients with significantly worse DSS. Positive viral status showed a correlation with improved DSS (*p* = 0.02) and tumors morphologically characterized by large cells were correlated with improved DSS as well (*p* = 0.03). Both presence of TILs and their brisk pattern of distribution were associated with better DSS (*p* = 0.003). No differences in survival were observed when assessing TILs only at the tumor periphery or also within the tumor stromal septa. By multivariate analysis (Fig. 2), increased Breslow thickness (*p* = 0.002) and stage (*p* = 0.026) (both evaluated as continuous variables) proved to be independent parameters associated to a shorter DSS. Considering the co-linearity of Breslow thickness and tumor size (Pearson’s correlation coefficient: 0.58), tumor size was introduced in a separate multivariate analysis and confirmed its statistical significance for DSS (*p* = 0.007). Finally, we found a strong independent correlation between brisk-TILs and improved MCC specific survival; conversely, the absence of TILs showed a negative correlation with DSS (*p* = 0.003). Viral status confirmed its prognostic role, being MCPyV + MCCs a subgroup with a significantly longer DSS (*p* = 0.011). Stratifying all cases into four distinct groups according to viral status and presence of a brisk pattern of TILs, the combination of negative viral status and absent/non-brisk TILs correlated with a significantly worse prognosis (*p* = 0.001), whereas patients with MCPyV + /brisk-TILs tumors showed a longer survival (*p* = 0.05). ALK expression was analyzed in respect to patient’s DSS both as a continuous variable based on staining intensity and as a discrete variable (negative *versus* positive and negative-weak *versus* intermediate-high), according to literature data [26]. No significant correlations with DSS were detected, neither in univariate nor in multivariate analysis.

By univariate analysis, shorter DFI was significantly associated with increased tumor size (*p* < 0.001), Breslow thickness (*p* = 0.013), deep extradermal invasion (*p* = 0.001) and vascular embolization (*p* = 0.05). Conversely, presence and a brisk pattern of TILs were associated with improved DFI (*p* = 0.033). By multivariate analysis controlling for the above-mentioned parameters with the inclusion of ALK expression and tumor stage, increased tumor size (*p* < 0.001) and progressive increase of ALK expression (*p* = 0.008) proved to be independent parameters associated with shorter DFI. As observed for DSS, Breslow thickness confirmed its independent prognostic value in a separate multivariate analysis (*p* = 0.007) (Fig. 2).

All data concerning DSS and DFI uni- and multivariate regression analyses are reported in Table 5

**Table 5:** Uni- and multivariate Cox regression analyses on disease-specific survival and disease-free interval

	***DSS***				***DFI***			
	***Univariate***		***Multivariate***		***Univariate***		***Multivariate***	
	**HR**	***p***	***HR***	***p***	**HR**	***p***	***HR***	***p***
Sex (female *vs. *male)	0.654	0.111			0.593	0.064		
Age (years)*	1.035	**0.011**	1.044	0.096	1.012	0.303		
Tumor site								
Head and neck (*vs*. other sites)	0.718	0.29			0.952	0.865		
Trunk (*vs*. other sites)	0.676	0.513			0.889	0.822		
Buttocks (*vs*. other sites)	1.292	0.468			1.292	0.449		
Extremities (*vs.* other sites)	1.268	0.396			0.923	0.775		
Unknown primary lesion (*vs. *known primary lesion)	0.97	0.959			0.862	0.837		
Tumor size (cm)*	1.37	**<0.001**	1.343	0.007**	1.41	**<0.001**	1.592	**<0.001**
Breslow thickness (mm)*	1.071	**0.003**	1.091	**0.002**	1.062	**0.013**	1.077	0.007***
Deep extradermal invasion (pres *vs. *abs)	3.085	0.063	3.105	0.077	6.549	**0.001**	2.12	0.21
Tumor stage*	1.287	**0.002**	1.294	**0.026**	1.145	0.126	1.128	0.324
Tumor edge (pushing *vs*. infiltrative)	1.392	0.353			1.425	0.314		
Growth pattern (organoid/trabecular *vs*. diffuse)	0.745	0.499			0.827	0.64		
Vascular invasion (pres *vs. *abs)	2.556	0.077	1.11	0.858	2.754	**0.05**	2.86	0.103
Skin ulceration (pres *vs. *abs)	1.794	0.084			1.199	0.614		
Necrosis (pres *vs. *abs)	1.453	0.221			1.307	0.352		
Cell type								
Conventional	0.85	0.573			0.906	0.717		
Spindle cell	1.532	0.229			1.953	0.906		
Large cell	0.365	**0.032**	0.586	0.49	1.002	0.994		
Pleomorphic	1.716	0.594			1.337	0.752		
Tumor infiltrating lymphocytes*	0.493	**0.003**	0.396	**0.003**	0.617	**0.033**	0.79	0.422
(abs, non-brisk, brisk)								
Mitoses/mm2*	0.999	0.902			0.995	0.672		
Solar elastosis (abs,1,2,3)*	0.931	0.636			0.879	0.369		
Proliferation index*	1.018	0.135			0.999	0.887		
Viral status (positive *vs. *negative)	0.427	**0.022**	0.367	**0.011**	1.075	0.821		
ALK expression*	0.969	0.852			1.204	0.201	1.805	**0.008**
ALK expression (positive *vs. *negative)	1.03	0.943			1.55	0.356		

*Abs* absent, *Pres* p resent; *DSS* disease-specific survival; *DFI* disease-free interval

*Continuous variables; **Considering tumor size instead of Breslow thickness in the multivariate analysis; ***Considering Breslow thickness instead of size in multivariate analysis

### Clinical-Pathological Comparison Between MCC of Unknown Primary Lesion and MCC with Known Primary Skin Lesion

When comparing MCC-UP to MCC with known primary cutaneous lesion (excluding patients who presented with stage IV at diagnosis), MCC-UP patients showed a significantly lower mean age at diagnosis (65 *vs*. 75 years; *p* = 0.026). Apart from a greater prevalence of necrosis in the MCC-UP subgroup (*p* = 0.047), no further significant differences were observed between the two cohorts in respect to morphological features, viral status and ALK expression, as well as patient’s survival (Table 2).

### MCC Incidence Rate

**Fig. 3:**
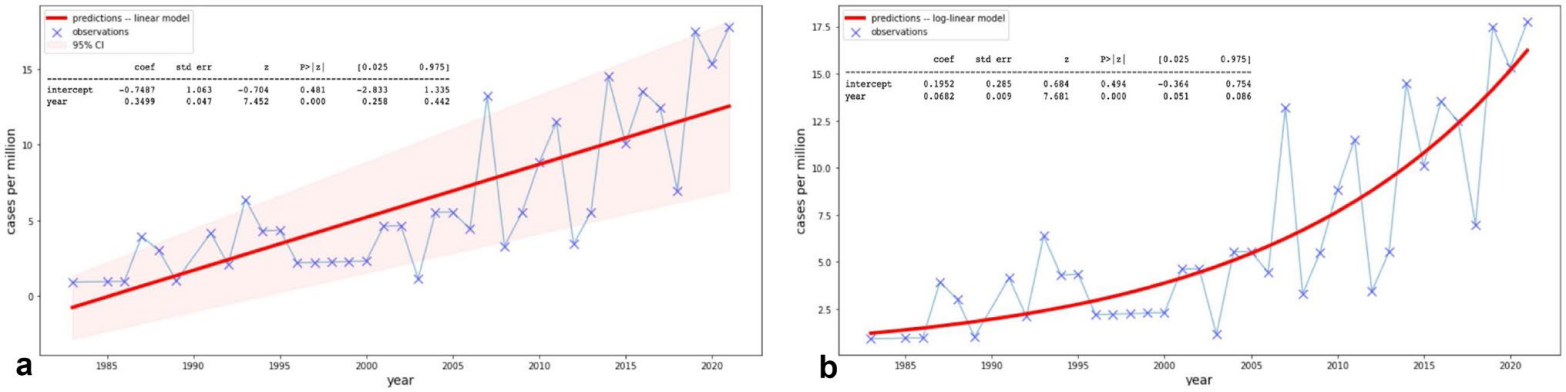
Both a linear model (**a**) and a generalized linear model (**b**) demonstrate an increase in MCC diagnoses from 1980 to 2021 in the city of Turin

Statistical analysis confirmed an exponential trend of the observed increase in MCC incidence for both a Linear Model (LM) and a Generalized Linear Model (GLM) fit with logarithmic transform, with *R*2 coefficients of 0.72 and 0.61, respectively. The LM estimated a yearly increase in the number of diagnosed cases per million inhabitants of 0.35, with estimated 95%-confidence intervals between 0.26 and 0.44 (*p* < 0.001) (Fig. 3). The GLM fit with logarithmic link function showed a similar significance (*p* < 0.001), relative to the yearly coefficient. The potential causes and dynamics of this trend were further explored through an APC analysis. A peak in MCC diagnoses in patients born between 1930 and 1950 (cohort effect) was observed, entangled with a peak among the age group ranging from 70 to 80 years. The increase in diagnoses in the last 20 years was shared among all age groups over 55 years, with a greater increase in patients aged 75 to 85 years, while the incidence rate among younger age groups (35 to 55 years) remained largely constant. Finally, the rate of incidence increase appeared to be slowing in recent calendar periods. It should be noted that no major population changes, nor relevant changes in the catchment areas of the considered hospitals were recorded in the last decades.

## Discussion

This paper represents one of the largest studies to date integrating clinical-pathological and phenotypical features of MCC with patient’s outcome and investigating MCC incidence trends in a single-city cohort.

Through the analysis of MCC diagnoses from 1980 to July 2021 in three of the main hospitals of the city of Turin (including two University Hospitals and one reference center for neuroendocrine pathology), we demonstrated a significant increase of MCC incidence. Specifically, an estimated yearly increase of MCC diagnoses of 3.5 per 100.000 inhabitants was observed, in accordance with what recently reported by Jacobs et al. in the USA [4]. Consistent with previously published reports [32], this increase was shared among all age groups over 55 years and was prevalent in patients aged 75–85 years. We believe that age is likely the main determinant of this growing rate, as suggested by the significant increase in mean life expectancy in past years (from 77 and 69.6 years in 1980 to 85.6 and 81.2 years in 2019 for females and males, respectively, according to the Italian Institute of Statistics data [33]). It could be speculated that an increase in MCC incidence may also be partly due to a shift of patients from minor centers to major hospitals of the city but we do not believe that this is the cause, as “Città della Salute e della Scienza Hospital” has always been a reference center for rare neuroendocrine pathology, including MCC. Among other putative factors, unprotected intentional sun exposure may certainly play a role [34, 35]: it can be assumed that in future years the evolving awareness of the risks of unprotected sun exposure may lead to a relative reduction of non-virus related MCC, which could conversely be counteracted by an increase in immunosuppression/immunosenescence-related MCCs. In fact, projection analyses estimate that MCC incidence will most likely continue to rise [4, 5], underscoring the continued need for early detection and optimal patient’s management.

MCCs of the current series presented as a dermal malignancy with frequent extension to the subcutis, displaying a prevalent trabecular/organoid pattern of growth with pushing margins, high mitotic index, extensive vascular embolization and a largely typical cytology showing small round uniform nuclei with scant cytoplasm, as extensively reported in literature [11, 36, 37]. Despite its characteristic morphology, MCCs needs to be distinguished from its mimickers, and the combination of CK20 and neuroendocrine marker expression, such as CGA and synaptophysin, has traditionally represented the first-line immunohistochemical approach in order to confirm the diagnosis. INSM1, one of the most recently introduced biomarkers of neuroendocrine differentiation, was extensively expressed in our series (100%) with a predominantly strong and diffuse nuclear reaction. Considering its high sensitivity [38] and easier microscopic evaluation, INSM1 could be an optimal and strongly reproducible tool for MCC diagnosis, and could therefore be used as a single neuroendocrine biomarker in addition to CK20, reducing both costs and time of evaluation.

Our analyses confirm and validate in a large series the prognostic value of clinical and morphological parameters such as tumor size, tumor thickness and stage in respect to DSS and DFI [13, 15, 18, 39–41]. In addition, male sex, tumor size, stage, deep extradermal invasion and vascular embolization were all parameters conferring an increased risk of disease progression. A significantly longer DSS and DFI in patients with TILs was observed, which further improved with a brisk pattern of infiltration, and was independent of viral status, tumor stage and size in DSS. The correlation between TILs and MCC survival has been widely demonstrated in the literature with a documented positive prognostic impact of increasing levels of CD3 + and CD8 + lymphocytes both in the intratumoral environment [17, 42] and at the tumor periphery [43–45]. Although the issue is still controversial and some studies reported TILs being associated with a poorer prognosis [46] the vast majority of published data strongly support the crucial role of the tumor immune microenvironment in controlling cancer growth, mirrored by its increased incidence and more aggressive course in immunocompromised individual and its sensibility to treatment with immune checkpoint inhibitors [47]. To date, standardized guidelines concerning evaluation of TILs are still lacking, and there is no consensus in whether to focus their evaluation exclusively at the tumor periphery or also in the intratumoral environment. We here suggest to asses TILs both at the tumor-stromal interface and within the tumor septa and to differentiate them as brisk and non-brisk, since this stratification proved to be of prognostic value. It should however be noted that a phenotypical and qualitative assessment of the T-cell infiltrate would provide a more precise prognostic stratification. In fact, T-cell infiltrates enriched for T cells reactive to tumor antigens have been correlated with a lower risk of metastasis and a lower stage, even among patients with low density infiltrates [48, 49].

In agreement with literature data on MCPyV prevalence ranging from 40 to 80% [36], in our cohort 69% of MCCs were MCPyV + . Positive viral status was a robust prognostic indicator associated with better DSS, irrespective of patient’s age, tumor size, thickness, stage, and TILs. Although data of the last ten years have shown controversial results [50–58], our findings are consistent with two of the largest recently published studies, which reported an improved outcome in MCPyV + MCC and a higher risk of recurrence, progression and death in MCPyV- MCC [12, 13]. Additionally, MCPyV + MCCs were significantly associated with younger age at diagnosis, absence of histological ulceration and lower mitotic rate, underlining a less aggressive phenotype compared to UV-related tumors [13, 50, 59, 60]. Such histological parameters should therefore be taken into account in the morphological evaluation of MCC. Interestingly, stratifying MCC according to viral status and TILs, the combination of negative viral status and absent/non-brisk TILs identified a subgroup of patients with significantly worse DSS. This original observation could therefore represent an easy-to-apply prognostic tool in order to address patient’s management.

Supporting the role of UV-induced damage in MCC, tumors located in the head and neck sun exposed skin were more frequently MCPyV-, whereas all MCCs localized on the buttocks were MCPyV + . Coherently, MCPyV- MCCs showed a significantly increased amount of solar elastosis in tumor adjacent dermis [59, 61]. Large nuclear size was significantly associated with positive viral status, whereas presence of significant cytological pleomorphism and of anaplastic giant tumor cells were exclusive features of MCPyV- MCCs. This is in line with emerging reports delineating cytological differences between MCPyV + and MCPyV- tumors, with the former being more frequently characterized by cells with uniform round and large nuclei and scant cytoplasm, and the latter by greater cytological heterogeneity and atypia [55, 56, 62, 63]. This could possibly reflect the differences in the genomic complexity of the two subgroups, where the high mutational burden in UV-induced MCCs probably accounts for the greater morphologic heterogeneity of the neoplastic cells.

ALK immunohistochemical reaction was observed in 87% of our cases; a significant correlation between ALK expression and viral status was detected, with MCPyV + MCCs being significantly more likely to show positive and higher ALK expression compared to MCPyV- cases. The expression and role of ALK in MCC is of emerging interest: only few reports have highlighted a positive correlation between ALK and viral status [13, 20, 29, 59], although the underlying mechanism has not been clarified, yet [22]. Along with our results, this suggests that MCPyV infection could play a role in promoting ALK overexpression in neoplastic cells [26]. Interestingly, we found increasing ALK tumor expression to be an independent indicator of progressively shorter DFI by multivariate analysis, suggesting strong ALK expression to be associated with a more aggressive clinical course. Similar prognostic observations have been reported in several solid tumors [64–67]. We were surprised to notice the opposite finding in a recent paper by Jaatinen et al., who observed a better survival in patients showing moderate/high ALK and phosphorylated ALK (p-ALK) tumor expression [26]. Such divergent results could be related to the use of a tissue microarray-based technical procedure in Jaatinen’s study [26] and/or to tumor heterogeneity which requires further investigations, in order to open the way to novel therapeutic approaches [68].

Fourteen of our cases presenting with macroscopic nodal disease lacked any evidence of a skin primary tumor (MCC of unknown primary, MCC-UP), and presented with a mean age at diagnosis of 65 years, which was significantly lower compared to the age at diagnosis of MCC with known primary skin lesion. This is in line with previous reports observing a similar mean age at diagnosis [69, 70] as well as a lower median age in the MCC-UP subgroup [71]. In apparent contrast with our results, no significant differences in demographics of MCC-UP was reported in the largest published series, but age was investigated considering a cutoff of 65 years (and not as a continuous variable), thus possibly explaining our diverging results [72]. Given the well-known controversy concerning the origin of these tumors, our result could possibly argue against the hypothesis of MCC-UP being a primary lymph node tumor, rather favoring a metastasis from an occult skin primary. In fact, a younger age at diagnosis may indicate a stronger and more efficient immune response that could lead to the regression of a primary skin lesion. This is also supported by recent observations assessing MCC-UP patients have lower rates of immunosuppression, higher likelihood of response to checkpoint inhibitions or immunologic therapy, and significantly improved survival as compared to stage IIIB MCC with known primary cutaneous lesion [70, 72, 73]. Nevertheless, in a recent review, rare cases of MCC-UP arising in the setting of immunosuppression have been described and found to be prevalently characterized by a negative viral status and worse prognosis [74]. It remains however unlikely that such tumors actually arise as a primary lymph node disease; in fact, evidence of UV-mutational signatures and high mutational burdens have been extensively reported in MCPyV- MCC-UP, both in immunocompetent and immunosuppressed patients [71–73].

## Conclusion

In conclusion, while confirming the increase of MCC incidence also in a region of North-Western Italy, our study underlines:

1. the high sensitivity of INSM1, which could be used as single diagnostic neuroendocrine biomarker in both skin and lymph node primary MCC, together with the expression CK20;
2. morphological evaluation of MCC could be restricted to tumor size and thickness as well as to a quantitative evaluation of TILs (with either brisk or non-brisk profiles), as they proved to be independent prognostic parameters;
3. positive viral status identifies a subgroup of patients with longer DSS (especially when combined with a brisk TILs profile): therefore, such parameter should not be missing in pathological reports;
4. ALK expression occurs in 87% of MCC and it is associated with positive viral status. Although still controversial, it could represent a future prognostic/predictive biomarker, but its role needs to be validated in larger multi-centric series.
